# Biomechanical adaptations enable phoretic mite species to occupy distinct spatial niches on host burying beetles

**DOI:** 10.1098/rspb.2024.0230

**Published:** 2024-03-20

**Authors:** Syuan-Jyun Sun, Simon Chen, Walter Federle, Rebecca M. Kilner

**Affiliations:** ^1^ Department of Zoology, University of Cambridge, Downing Street, Cambridge CB2 3EJ, UK; ^2^ International Degree Program in Climate Change and Sustainable Development, National Taiwan University, Taipei 10617, Taiwan

**Keywords:** attachment, biomechanics, burying beetles, competition, niche partitioning, phoretic mites

## Abstract

Niche theory predicts that ecologically similar species coexist by minimizing interspecific competition through niche partitioning. Therefore, understanding the mechanisms of niche partitioning is essential for predicting interactions and coexistence between competing organisms. Here, we study two phoretic mite species, *Poecilochirus carabi* and *Macrocheles nataliae* that coexist on the same host burying beetle *Nicrophorus vespilloides* and use it to ‘hitchhike’ between reproductive sites. Field observations revealed clear spatial partitioning between species in distinct host body parts. *Poecilochirus carabi* preferred the ventral side of the thorax, whereas *M. nataliae* were exclusively found ventrally at the hairy base of the abdomen. Experimental manipulations of mite density showed that each species preferred these body parts, largely regardless of the density of the other mite species on the host beetle. Force measurements indicated that this spatial distribution is mediated by biomechanical adaptations, because each mite species required more force to be removed from their preferred location on the beetle. While *P. carabi* attached with large adhesive pads to the smooth thorax cuticle, *M. nataliae* gripped abdominal setae with their chelicerae. Our results show that specialist biomechanical adaptations for attachment can mediate spatial niche partitioning among species sharing the same host.

## 1. Introduction

Niche theory, a fundamental concept in ecology, proposes that species coexistence is facilitated by the differentiation of distinct niches, allowing animals to avoid direct competition for resources [1]. This theory is pivotal for elucidating the roles of different species and the structure of ecological communities. In symbiotic relationships, where organisms have a closely intertwined existence, niche partitioning becomes particularly critical. Different species of symbiont often inhabit the same host species, potentially resulting in interspecific competition over limited resources or space [2,3]. This is especially true for phoretic organisms (phoronts), which use their host as a vector for dispersal and temporarily attach to its surface [4]. The interactions between phoretic species on their hosts, therefore, represent a unique opportunity to analyse the coexistence mechanisms underlying spatial niche partitioning.

Environmental heterogeneity has been hypothesized to be one of the major drivers of niche partitioning [5], causing competing species to diverge and specialize in different resources or habitats, both spatially and temporally [5–7]. Specialization is associated with divergent adaptations in competing species to optimally use different niches. Phoronts may behaviourally adjust their distribution on the surface of a host, depending on variation in host morphological characters, phoront density and the extent of interspecific competition [8]. Competition between coexisting species is largely determined by the extent of overlap of their distribution which, in turn, is mediated by the population size of each species [9]. Each species may occupy a preferred site starting at low population densities, but be forced to move to a less preferred location at high densities [10], where it is more likely to encounter the rival species. Although the importance of niche partitioning between coexisting symbiotic organisms has often been highlighted [11,12], few experimental studies have investigated the relative importance of intra- and interspecific competition, or analysed adaptations for niche partitioning.

**Figure 1.**
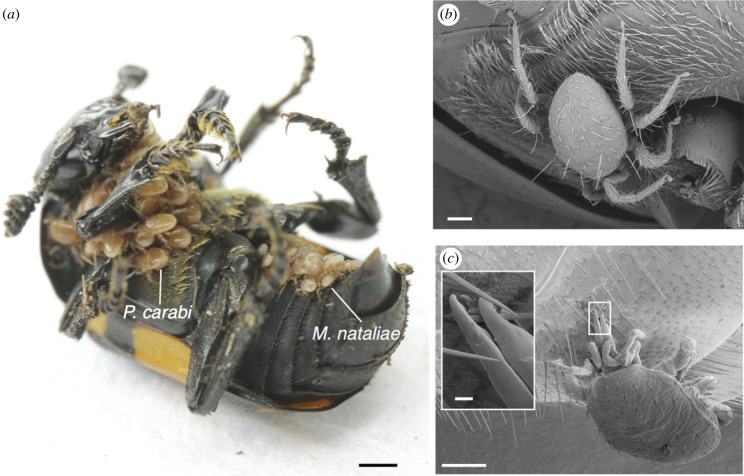
(*a*) Mite coexistence on the ventral side of burying beetles (*N. vespilloides*). (*b*) *P. carabi* deutonymph attaches to the *N. vespilloides* thorax, whereas (*c*) adult females of *M. nataliae* primarily occupy the abdomen. Scale bars: (*a*) 1 mm, (*b*) 200 µm, (*c*) 200 µm (inset 20 µm).

Burying beetles carry with them at least 14 species of mites (belonging to four families: Parasitidae, Macrochelidae, Uropodidae and Histiostomatidae) [13]. At our study site in Cambridgeshire, UK (see Methods), *Poecilochirus carabi* and *Macrocheles nataliae* are two mite species commonly associated with *Nicrophorus vespilloides* burying beetles (figure 1*a*). Burying beetles breed upon small vertebrate carrion. Once the beetles locate a carcass, they prepare it into an edible nest for their offspring by removing any feathers or hairs, rolling it into a ball and burying it underground [14]. The larvae then hatch from eggs laid near the carcass and crawl to the prepared nest, upon which they feed, aided by the parents [15]. Just like the beetles, mites also require a carcass to breed upon [16]. However, mites are ‘phoretic’, relying on the beetles to transport them between carcasses. Burying beetles, and the mite community they transport, present an ideal system to study the evolution of niche specialization and the underlying mechanisms of niche partitioning. There is limited space for mite attachment on the beetle's body surface, leading to competition for attachment sites. However, as different beetle body parts differ in their surface texture, this heterogeneity potentially allows the mites to exploit distinct spatial niches, clinging to the areas best suited to them. In any case, mites are likely to experience strong friction or drag forces from different directions when they travel with their hosts, especially when the beetles move through the soil or fly to search for carcasses.

In this study, we tested whether *P. carabi* and *M. nataliae* indeed occupy different spatial niches on their burying beetle host, and studied the mechanisms behind such spatial niche partitioning. We started by examining the natural occurrence and density of the mites on field-collected beetles. To test for within-host competition and any spatial preference of the mites, we experimentally manipulated the density of both mite species under laboratory conditions. We used scanning electron microscopy to characterize mite attachment devices and different beetle body parts, including their surface roughness and hairiness, which can influence mite attachment. Finally, to test whether the two mite species had specialized in their ability to attach to different body regions of the host, we measured the mites' attachment forces and identified their mechanism of attachment at their preferred location on the beetle.

## 2. Methods

### (a) Study site and study organisms

Field trapping was carried out between August and October in 2018, covering the breeding season of *N. vespilloides* in Cambridgeshire. Five hanging traps at each study site were all baited with a fresh mouse carcass, and were set up in two woodlands in Cambridgeshire, UK: Gamlingay (latitude: 52.15555°; longitude: −0.19286°) and Waresley (latitude: 52.17487°; longitude: −0.17354°). Traps were checked every 2–3 weeks and the bait was replenished. Our field trapping showed that *P. carabi* and *M. nataliae* were routinely found on *N. vespilloides* at our study sites (figure 1*a*). Another mite species, *Poecilochirus subterraneus*, was also found to associate with *N. vespilloides* but only on 4 out of 326 individuals. Therefore, we focused our laboratory experiments on the two most commonly found mite species, *P. carabi* and *M. nataliae*. *Poecilochirus carabi* attaches to adult beetles as deutonymphs [17], whereas *M. nataliae* attaches as adult females [18]. Upon the beetle's arrival at a carcass, *P. carabi* moult into the adult males and females to mate, whereas *M. nataliae* are haplodiploid and a fertilized female can start breeding immediately [19,20]. Once the beetle's breeding is completed, most of the offspring will attach to the adult beetles for the purpose of dispersal. The mean individual mass of *P. carabi* deutonymphs and adult female *M. nataliae* was 256 ± 26.7 µg (mean ± s.d.; *n* = 36) and 104 ± 20.1 µg (mean ± s.d.; *n* = 20), respectively. The mass of individual mites was measured using a MC5 microbalance (Sartorius, Goettingen, Germany).

### (b) Field observation: distribution of mites over the beetle's body surface

From field-collected beetles, we visually examined the mites and determined mite prevalence (the proportion of beetles that harboured mites) and mite abundance (the number of mites carried by infested beetles). The entire body surface of each captured beetle was checked thoroughly for the presence of mites under a stereo-microscope. All mites were removed for counting. We determined the number of each mite species per beetle, and on each body region of the beetle (head, ventral thorax, ventral abdomen, pronotum and elytra). The beetle's body size (measured as the width of the pronotum) and sex were also determined.

To assess differences in spatial preference between *P. carabi* and *M. nataliae*, we used chi-square tests to evaluate whether or not the number of mites attached to specific beetle body parts followed a random distribution (with uniform density). All 6020 and 38 individuals of *P. carabi* and *M. nataliae* were pooled. The observed number of mites was compared with the number expected from the relative surface area of each body part. The beetles' body surface area was approximated as the sum of the projected areas of the body in dorsal and ventral view, measured from images using image analysis software (ImageJ, http://imagej.nih.gov/ij/). As each body region is located either on the ventral or dorsal side of the body, their surface areas were also measured from images taken in dorsal or ventral view. Note that this approach reduces the insect's three-dimensional surface topography to a two-dimensional estimate and will hence underestimate the true surface area. However, due to beetles' horizontally flattened body geometry, this approach is fully adequate to measure the relative size of the different body regions. We acquired high-resolution images of the beetles’ dorsal and ventral sides using a macro-lens (electronic supplementary material, figure S1). The images were imported into ImageJ software, and outlines of the whole beetle and each body part were manually selected to measure the area. We then predicted the number of mites by multiplying the total number of mites by the proportion of surface area of each body part, for each mite species. For example, if there were 50 mites on a beetle and the thorax accounted for 20% of the total body surface, then we predicted there should be 10 mites on the thorax, assuming that they were evenly distributed over the body surface.

### (c) Maintenance of the beetle and mite colonies

Beetles and mites collected from the field study sites were used to establish laboratory colonies, which were maintained as described elsewhere [21,22]. Sexually mature beetles (2–3 weeks after adult emergence) were used for experiments. A stock of each mite species was maintained by allowing pairs of beetles to breed once a month on mouse carcasses alongside 15 mites (*n* = 10). All beetles and mites were maintained under laboratory conditions at 21 ± 2°C with a 16 : 8 h light : dark photoperiod. As beetle sex did not influence mite preference for body parts in field-collected beetles (generalized linear mixed model (GLMM), *P. carabi*: *χ*^2^ = 0.15, d.f. = 1, *p* = 0.701; *M. nataliae*: *χ*^2^ = 0.58, d.f. = 1, *p* = 0.446), we did not expect beetle sex to determine the mites' attachment performance. Therefore, only female beetles were used in further laboratory experiments.

### (d) Within-host competition experiment

To investigate the relative importance of intra- and interspecific competition in determining the site of mite attachment, we experimentally transferred different-sized groups of *P. carabi* and *M. nataliae* onto mite-free beetles that had not encountered any mites previously. We began by testing the role of intraspecific competition. We initially manipulated the density of the focal species in the presence of one mite from the other mite species, as the default for testing the effects of intraspecific competition. When the focal species was *P. carabi*, individual mites were sequentially introduced to achieve groups of 1, 5, 10, 25 and then 50, while one *M. nataliae* mite was present on the beetle (*n* = 10 beetles for each density combination). When the focal species was *M. nataliae*, we created groups of 1, 3, 5, 10, 25, 50 individuals that were present alongside a single *P. carabi* mite (*n* = 10 beetles for each density combination). These ranges of abundance captured the natural mite abundance for *P. carabi* (0–276, a mean abundance per beetle of 18.5 ± 1.7, median = 10, and 8% carry more than 50 mites). *Macrocheles nataliae* exists at lower densities in nature (typically 0–5 mites per beetle). *Macrocheles nataliae* was always introduced prior to the introduction of *P. carabi* because *M. nataliae* was less mobile than *P. carabi* and because it was logistically impossible to introduce both mites simultaneously. In each trial, both the beetle and the mites were placed together at the bottom of a transparent container (2 × 2 × 2 cm). Then, the mites were given 5 min to find the beetle, climb onto it and choose a spot to attach themselves. This time window was sufficient for all mites to complete their attachment. We used two digital cameras with a macro-lens (Imaging Source DMK 23UV024 with 1 inch (approx. 25 mm) focal length lenses) to take photos of the dorsal and ventral side of the beetles for mite counting (electronic supplementary material, figure S1). We then recorded the number of mites of each species and their attachment site. All beetles and mites were only used once for each trial through the whole experiment.

The data were analysed using a generalized linear mixed model (GLMM) with a binomial distribution, with the beetle's ID included as a random factor. To investigate intraspecific competition within each mite species, we analysed the proportion of mites on different beetle body parts by creating a two-column response vector (i.e. the number of mites that was on the focal body part and the number of mites that was not on the focal body part) using the *cbind* function. As explanatory variables, we included the total density (total number of mites divided by body surface area) of the focal mite species as a continuous variable, and beetle body part as a categorical factor. We analysed the data for *P. carabi* and *M. nataliae* separately.

To examine the role of interspecific competition at different densities of the focal mite species, we manipulated the abundance of both species simultaneously. Specifically, 1, 5, 10, 25 or 50 mites of *P. carabi* were combined with 1, 3 or 5 mites of *M. nataliae*. For each of these density combinations, the sample size was *n* = 10. These results were analysed as before using a GLMM. To investigate the effect of interspecific competition on mite distribution, we analysed how the density of each mite species on different body parts depended on the total density of the other species. Explanatory variables included beetle body part as a categorical factor, the total density of *P. carabi*, and the total density of *M. nataliae*, and their interaction with body part. The beetle's ID was included as a random factor.

### (e) Reproductive output of *P. carabi* and *M. nataliae* mites

To investigate whether the two mite species differed in reproductive output, we gave unrelated burying beetle pairs (2–3 weeks old) either 10 *P. carabi* mites or 10 *M. nataliae* mites to breed alongside. We added the same number of individuals for each mite species so we could assess which species was intrinsically more fecund. In total, reproductive output was measured in 2016–2017 in 63 and 40 broods of *P. carabi* and *M. nataliae*, respectively, across three different blocks.

Breeding took place on a 7–15 g mouse carcass in a plastic container (17 × 12 × 6 cm filled with 2 cm of moist soil). After the dispersal of beetle larvae into the soil to pupate, we harvested the mite offspring that were attached to the adult beetles. The beetles were anaesthetized with CO_2_ and then mites were removed from beetles with a fine brush and tweezers.

To investigate whether the reproductive output differed between the two mite species, we included the total number of mite offspring as the response variable in this analysis. We included as an explanatory variable the mite species (*P. carabi*/*M. nataliae*) and carcass mass as a covariate. Since overdispersion was detected in the model, we analysed the total number of mite offspring with a GLMM and negative binomial distribution. We included block as a random effect since the experiment was carried out in three blocks.

### (f) Characterization of mites and burying beetle body regions by scanning electron microscopy

To characterize the mechanism of mite attachment on the beetles, we allowed mites to attach freely to freshly killed beetles (thawed after freezing), before flash freezing the mites on the beetle in liquid nitrogen. The mites mostly remained attached during this process. We analysed *P. carabi* and *M. nataliae* separately, at a density of 10 and 5 individuals, respectively. We studied these mite specimens using cryo-scanning electron microscopy with a Zeiss EVO HD15. We also imaged the surfaces of different body regions of the beetle (electronic supplementary material, figure S2; *n* = 4 beetles), and quantified differences in hair diameter (measured at the base) between the thorax and the abdomen. To quantify the differences in hair density between the thorax and the abdomen, we counted the number of hairs in each body part by randomly selecting three non-overlapping areas of 500 µm^2^. To control for non-uniformity of the types of surface within these body regions, the three areas were randomly selected over the entire thorax area. On the abdomen, we focused on the central regions of the sternites where the shorter hairs were scattered and most abundant. We chose this area because mites preferentially attached to the centre of sternites. To further investigate the number of different types of hairs on the abdomen, we counted the number of long and short hairs from three randomly selected regions in the anterior side of the 1st abdominal sternite. Each region was selected by controlling for width (500 µm^2^) but we allowed the length of the region to vary depending on how much space was available.

In subsequent experiments, we froze beetles (*n* = 5) carrying *M. nataliae* with liquid nitrogen, and removed the head and thorax of the beetles to allow viewing of *M. nataliae* head regions from additional angles (most mites stayed attached even when dead), followed by freeze-drying in a Quorum Emitech K775X freeze-dryer at −18°C. The specimens were then returned to room temperature in a desiccator to avoid condensation. We imaged these specimens using a FEI Verios 460 scanning electron microscope.

To investigate if the hairs on the burying beetle's thorax differed in thickness from those on the abdomen, we analysed the diameter at the hair base (log transformed) and hair density as response variables with a GLMM and Gaussian and Poisson distribution, respectively. Hair density was measured as the number of hairs within a cuticle area of 500 µm^2^. To investigate differences in the number of different types of hairs on the abdomen, we used a GLMM with Poisson distribution and hair type as an explanatory variable. Since hairs were repeatedly measured from the same individuals, we also included the beetle's ID as a random factor in these analyses.

### (g) High-speed video recording of the movements of mite attachment pads

To investigate how each mite species attached to beetles using adhesive pads, we filmed pad movement and attachment using a high-speed camera (Vision Research Phantom v7.1) at a frame rate of 1000 frames per second (fps) and a resolution of 600 × 800 pixel. We allowed mites to attach freely to freshly killed beetles and recorded them as they moved on different body parts. In addition, we quantified differences in adhesive pad contact area between *P. carabi* and *M. nataliae* by allowing mites to walk upside-down on glass coverslips. Steps of individual legs were recorded using coaxial illumination with the high-speed camera attached to a stereo-microscope. This illumination shows the pad contact zone in high contrast as a dark area on a bright background [23]; we recorded the maximum contact area of each recorded step.

To investigate differences in pad contact area between mite species, we used a GLMM with a Gaussian distribution; contact area (log transformed) was the response variable, and mite species (*P. carabi/M. nataliae*) the explanatory variable.

### (h) Measurement of mite attachment forces

We investigated the attachment performance of both mite species on different beetle body parts and artificial test substrates, at different pull-off angles. Using female beetles with ablated legs, we tested attachment to the ventral thorax, the ventral abdomen, the pronotum, and the elytra for normal (90° to substrate) and shear-dominated pulls (15° to substrate, pulling toward the posterior direction of the mite). We did not test pure shear (0°) in order to avoid entanglement of the thread with the beetle body. Additionally, we tested the mites' attachment to artificial substrates (Al_2_O_3_ lapping films with particles embedded in polyester with nominal asperity sizes of 0.05 µm, 0.5 µm and 16 µm, as well as smooth polyester film; UltraTec, CA, USA) in normal (90°), shear-dominated (15°) and pure shear (0°) directions.

Each mite was carefully glued by its dorsal shield to one end of a fine cotton thread (diameter: 0.2 mm) using cyanoacrylate glue under a stereo-microscope (Wild M8, 1× Plan objective), without impeding the movement of its legs. The thread was attached to a hook glued to the underside of a beam 15.5 mm × 7.5 mm × 0.15 mm (free length × width × thickness) of a custom-built fibre-optic 1D-force transducer mounted on a DC motor stage (M-126PD, Physik Instrumente, Karlsruhe, Germany) so that a motor-controlled pulling movement detached the mite from its substrate. The fibre-optic sensor measured the distance between its tip and a piece of reflective foil glued to the upper side of the beam (and hence the deflection of the beam); the voltage signal was converted to a force after calibration with weights. A custom Labview program (National Instruments, Austin, TX, USA) recorded the force, controlled the movement of the motor stage and triggered a synchronized video recording of the experiment [24].

For each force measurement, we allowed mites to attach to the body part/test substrate for 5 s before pulling at a constant speed of 500 µm s^−1^ (see electronic supplementary material, video S1). We analysed the force traces using Matlab (The Mathworks, USA). For logistical reasons, we tested each mite either on all beetle body parts or on all lapping film substrates. Thus, each mite was used for four measurements (beetles: thorax, abdomen, pronotum and elytra; substrates: 0.05 µm, 0.5 µm, 16 µm and smooth) in randomized order at one retraction angle. All measurements were conducted at a room temperature of 21°C. The summary of raw detachment forces at different angles on different substrates and beetle body parts can be found in electronic supplementary material, table S8.

**Figure 2.**
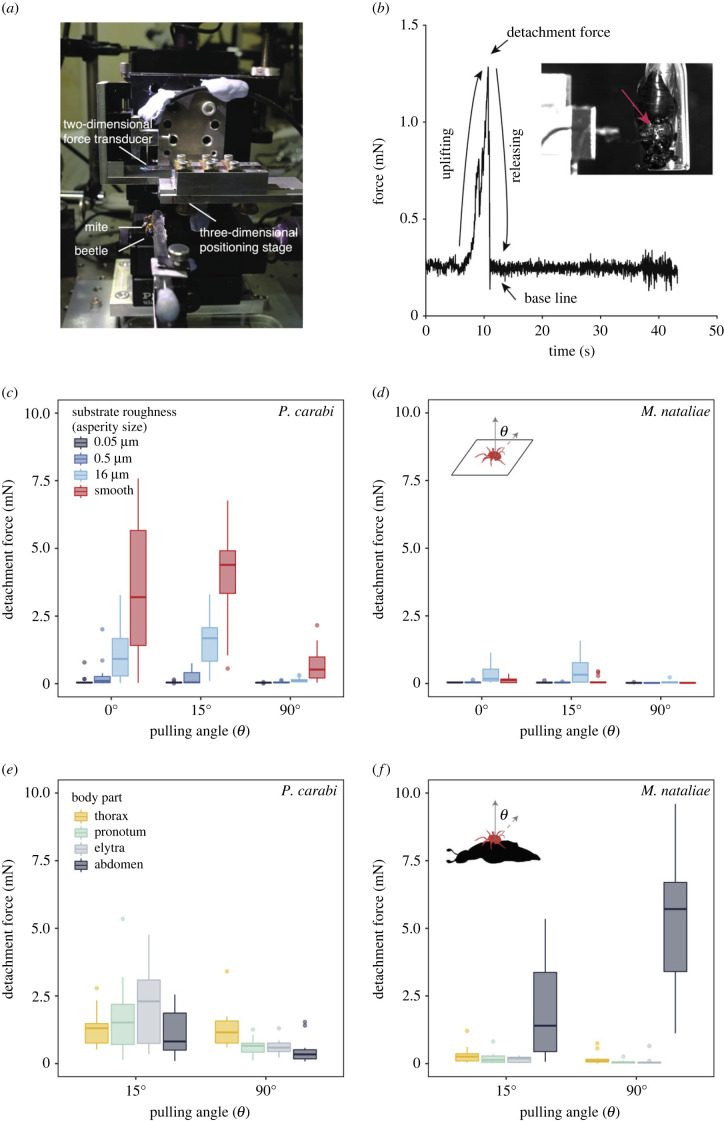
Force measurements on *P. carabi* and *M. nataliae* mites revealing specializations to different substrates and beetle body parts. (*a*) Experimental set-up and (*b*) an example of a recorded detachment force. The inset shows a *P. carabi* deutonymph (red arrow) holding onto the ventral side of the thorax of a beetle. Detachment forces at different pulling angles (*θ*) generated by (*c*) *P. carabi* and (*d*) *M. nataliae* on substrates with different roughness and by (*e*) *P. carabi* and (*f*) *M. nataliae* on different beetle body parts. Median values, 25th and 75th percentiles, and outliers are as shown. The whiskers denote 1.5 times interquartile range.

We analysed differences in the maximum detachment force (mN; log-transformed) between mites when attached to beetles or artificial substrates. For beetle attachment we included beetle body part (abdomen, elytra, pronotum and thorax), mite species (*P. carabi*/*M. nataliae*) and retraction angle (90° and 15°) as explanatory variables, whereas for substrate attachment we included substrate (0.05 µm, 0.5 µm, 16 µm and smooth), mite species and retraction angle (90°, 15°, 0°) as categorical factors. Additionally, individual ID of mites was included as a random factor since each mite was tested across all beetle body parts or substrate surfaces. We used separate models for each retraction angle (90° and 15° for beetle body parts, and 90°, 15°, 0° for smooth and rough substrates) since each mite received treatments within the same retraction angles.

### (i) Statistical analyses

All analyses were conducted in R (version 3.4.3) using GLMMs with the *glmer* function in the ‘lme4’ package [25]. Response variables were log transformed to meet normality and homogeneity of variance assumptions. *P* values were obtained with *Anova* function with type ‘III’ sum of squares in the ‘car’ package. Where a significant effect was detected, *post-hoc* Tukey honestly significant difference (HSD) tests were conducted for further pairwise comparisons between treatments using the ‘lsmeans’ package [26].

## 3. Results

### (a) Niche partitioning between mites on field-collected beetles

In total, 326 *N. vespilloides* beetles were collected from the field. Of these, 90.5% carried *P. carabi* (figure 1*b*) with a mean abundance per beetle (excluding mite-free individuals) of 20.4 ± 1.75 and a median of 9 (range 1–276; 91.2% carried <50 mites); 7.1% of beetles carried *M. nataliae* (figure 1*c*) with a mean abundance per beetle (excluding mite-free individuals) of 1.7 ± 0.23 and a median of 1 (range 1–5). A total of 22 out of 23 beetles carrying *M. nataliae* harboured *P. carabi*, whereas 22 out of 295 beetles carrying *P. carabi* also harboured *M. nataliae.* The mites were mostly found on the ventral side of the beetles (figure 1*a*).

**Figure 3.**
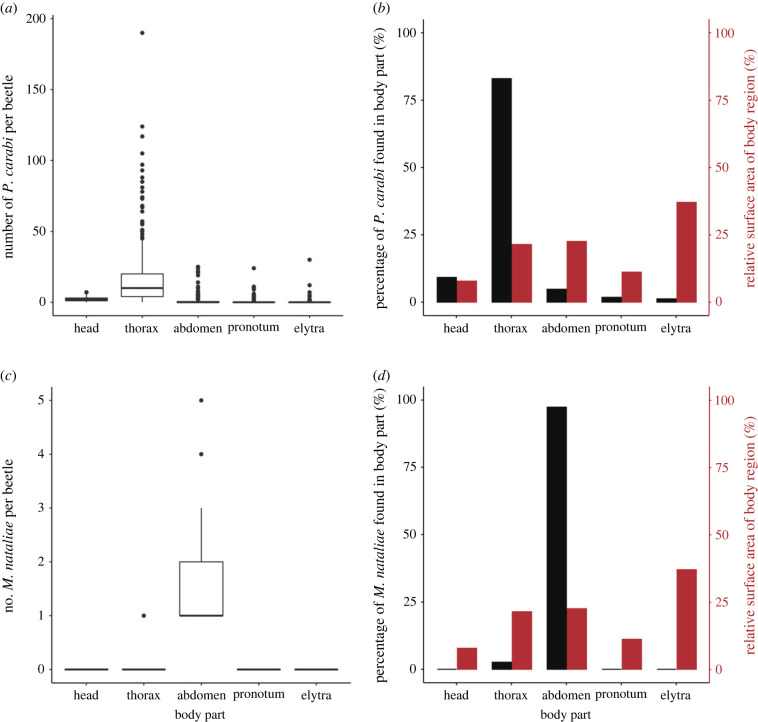
(*a,b*) Mean number and observed distribution of *Poecilochirus carabi* and of (*c,d*) *Macrocheles nataliae* mites present per beetle on each body part of field-collected *N. vespilloides* beetles. (*a,c*) Plots show medians (centre lines), interquartile ranges (boxes), and the largest and smallest values (whiskers) that are not outliers (circles). (*b,d*) Observed distribution of all collected mites (black bars) over the body parts, given as a percentage of the total number of mites collected, compared with the relative surface area of the beetle body parts (red bars). For all panels, ‘thorax’ refers to the ventral thorax region, ‘abdomen’ refers to the ventral abdomen. The pronotum is part of the dorsal thorax, and the elytra cover the dorsal abdomen.

We determined the number of each mite species on five body parts of the beetles (head, ventral thorax, ventral abdomen, pronotum and elytra), and compared it with the null distribution expected if mites simply distributed themselves randomly across these five body regions, with numbers proportional to surface area (see Methods). We found that the two mite species showed distinct aggregation patterns (electronic supplementary material, table S1). *Poecilochirus carabi* mainly attached to the beetle's ventral thorax (figure 3*a,b*), whereas *M. nataliae* attached almost exclusively to the beetle's ventral abdomen (figure 3*c,d*).

### (b) Experimental manipulations of intra- and interspecific competition

**Figure 4.**
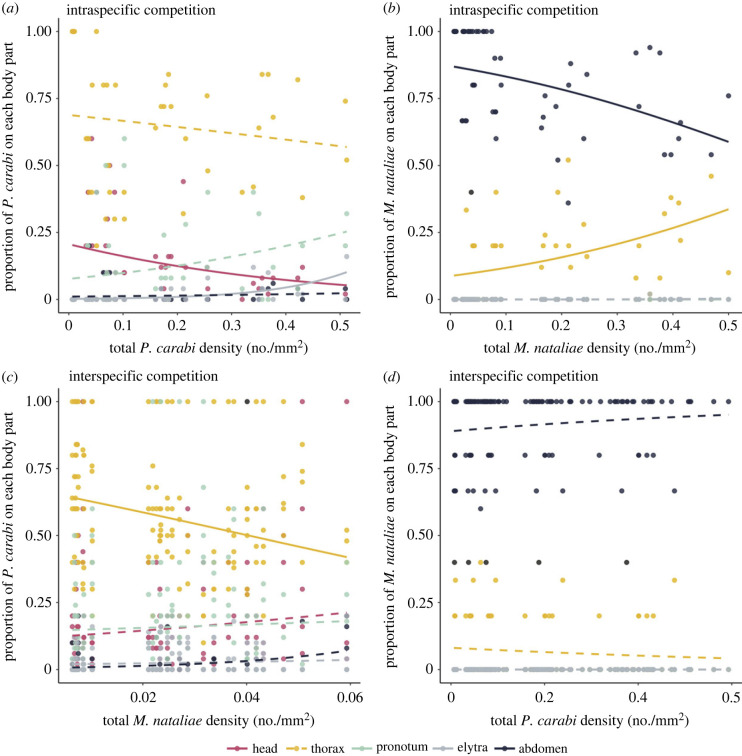
Proportion of *P. carabi* and *M. nataliae* on different beetle body parts when experimentally exposed to different levels of intraspecific competition (*a,b*) and interspecific competition with the other mite species (*c,d*). Proportion of mites are the number of mites of each body part divided by the total number of mites, whereas total densities represent the total number of mites per beetle divided by the total available surface area (in mm^2^). Solid lines are statistically significant regression lines from GLMMs, whereas dashed lines are not significant relationships. Note that in *M. nataliae*, the proportion on head, pronotum and elytra was zero.

To investigate the distinct roles of intra- and interspecific competition in determining the site of mite attachment, we manipulated the density of both *P. carabi* and *M. nataliae* on each beetle (see Methods). To isolate the effects of intraspecific competition, we initially manipulated the density of the focal species in the presence of a single member of the other species. (Since most beetles in nature carry at least one mite, we used a beetle plus one mite as our starting point for these manipulations.) Under these conditions, we found that the proportion of *P. carabi* on each body part was determined by the total *P. carabi* density on a beetle, though the effect differed between different body parts (figure 4*a*; electronic supplementary material, table S2). Specifically, we found *P. carabi* were most likely to attach to the beetle's ventral thorax, followed by the head, pronotum, and were consistently least likely to attach to the elytra and ventral abdomen. As we increased the total density of *P. carabi* on the beetle, the proportion of *P. carabi* on the thorax, pronotum and abdomen remained unchanged. However, there were proportionally fewer *P. carabi* on the head, and proportionally more *P. carabi* on the elytra. We found a contrasting pattern for *M. nataliae*, with total *M. nataliae* density influencing the proportion of attachment to each body part in different ways (figure 4*b*; electronic supplementary material, table S2). *Macrocheles nataliae* was mostly likely to attach to the beetle's ventral abdomen, followed by the ventral thorax, consistent with the observed patterns on field-collected beetles (see above). As overall densities increased, proportionally more *M. nataliae* shifted from the abdomen to the thorax. No *M. nataliae* were ever found on the head, pronotum and elytra.

To investigate the effect of interspecific competition at different densities of the focal mite species, we manipulated the abundance of both species simultaneously (see Methods). Our results show that interspecific competition had a different effect on the distribution of *P. carabi* and *M. nataliae*. Specifically, the proportion of *P. carabi* on the thorax decreased as *M. nataliae* density increased, whereas the proportion of *P. carabi* on the other body parts remained unchanged (figure 4*c*; electronic supplementary material, table S3). By contrast, there was no significant effect of *P. carabi* density on the distribution of *M. nataliae* (figure 4*d*; electronic supplementary material, table S3).

### (c) Laboratory reproduction of mites

To investigate whether *P. carabi* and *M. nataliae* differ in their reproductive output, we gave a mite-free burying beetle pair either 10 *P. carabi* or 10 *M. nataliae* mites to breed alongside (*n* = 63 burying beetle pairs exposed to *P. carabi*, and 40 beetle pairs exposed to *M. nataliae*). We found that at dispersal of beetle larvae *P. carabi* produced nine times more offspring than *M. nataliae* (GLMM, *χ*^2^ = 245.99, d.f. = 1, *p* < 0.001; electronic supplementary material, figure S3).

### (d) Mite attachment devices and cuticular surface of beetle body parts

**Figure 5.**
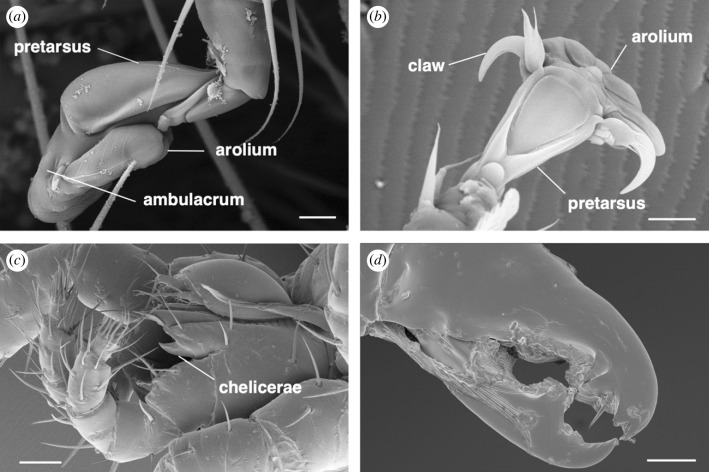
(*a*) *Poecilochirus carabi* mites lack claws and attach with a cushion-like, enlarged arolium. (*b*) *M. nataliae* possess distinct claws with an arolium to recruit additional forces. (*c*) Chelicerae of *P. carabi*. Note the small mite (neither of the mite species in this study) above the chelicera*.* (*d*) Chelicera of *M. nataliae* which generate a strong attachment by gripping setae on the beetle's abdomen (see inset in figure 1*c*). Scale bars: (*a*) 10 µm, (*b*) 10 µm, (*c*) 50 µm, (*d*) 15 µm.

*Poecilochirus carabi* bears an extensible arolium at the end of the elongated pretarsal ambulacrum (terminology following [27]), but no visible claws (figure 5*a*). By contrast, *M. nataliae* has an arolium flanked by two claws (figure 5*b*). Using high-speed video to record the tarsi of mites walking upside-down on glass, we compared the contact area of pads between *P. carabi* and *M. nataliae* (see Methods). *Poecilochirus carabi* arolia had a slightly larger contact area (664 ± 300 µm^2^; mean ± s.d.; *n* = 43 contacts) than *M. nataliae* (505 ± 113 µm^2^; mean ± s.d.; *n* = 22 contacts) (GLMM, *χ*^2^ = 5.57, d.f. = 1, *p* = 0.018), but contact area per body mass was higher in *M. nataliae* (505/104 = 4.85 versus 664/256 = 2.59 µm^2^ µg^−1^).

Our high-speed recordings of the tarsi of mites attaching to the body surfaces of beetles showed that *P. carabi* can use their arolia not only to attach to smooth surfaces, but also to cling to individual beetle hairs, by deforming and ‘wrapping’ the arolium around the hairs (electronic supplementary material, movie S4). *Macrocheles nataliae* mites were also able to attach to the beetle hairs but in a completely different way: they gripped them firmly with their dentate pincer-like chelicerae (pairs of mouthpart appendages). Unlike *M. nataliae*, *P. carabi* did not use their edendate chelicerae for attachment (figure 1*c* and figure 5*c*,*d*).

Scanning electron microscopy revealed that the cuticle surface of all beetle body parts (electronic supplementary material, figure S2) on the head, pronotum and elytra was smooth or weakly patterned and interspersed with shallow protrusions or indents (electronic supplementary material, figure S2A–C; possibly campaniform sensilla), or, on the thorax and abdomen, with hairs (electronic supplementary material, figure S2D–F; likely mechano- or chemosensory sensilla). The hairs on the thorax were thinner than those on the abdomen (hair diameters, thorax: 9.36 ± 2.27 µm, *n* = 234; abdomen: 11.15 ± 2.24 µm, *n* = 749, mean ± s.d.; GLMM, *χ*^2^ = 107, d.f. = 1, *p* < 0.001). Not only the thickness of hairs but also their distribution on the surface of the beetle's body differed between the ventral thorax and ventral abdomen. On the ventral thorax, the hairs were relatively uniform (electronic supplementary material, figure S2D), whereas two distinct types of hairs occurred on the abdomen (electronic supplementary material, figure S2E,F). The longer hairs were found on the anterior side of the 1st abdominal sternite, and at the posterior end of each abdominal sternite (electronic supplementary material, figure S1E), whereas the shorter hairs were scattered across the middle area of each sternite (figure 1*c*; electronic supplementary material, figure S2F). These short hairs occurred with a 2.6 times higher density than the long hairs on the abdomen (GLMM, *χ*^2^ = 523.2, d.f. = 1, *p* < 0.001), and 2.0 times higher density than the long hairs on the thorax (GLMM, *χ*^2^ = 195.9, d.f. = 1, *p* < 0.001).

### (e) Attachment force measurements of living mites on artificial substrates

To characterize the mites' attachment performance, we tested them on substrates of different roughness before testing their attachment to the beetles themselves (figure 2*a,b*, Methods). We also varied the direction of the pulling force, including lower angles to simulate the forces experienced by mites travelling on beetles in flight or walking through soil.

We found a significant effect of substrate roughness on the detachment forces of both mite species, but the effects varied depending on mite species and substrate angle (GLMM, substrate × species: *χ*^2^ = 146.65, d.f. = 3, *p* < 0.001; GLMM, substrate × angle: *χ*^2^ = 39.64, d.f. = 6, *p* < 0.001; figure 2*c,d*; electronic supplementary material, table S4). In general, *P. carabi* attached better on smooth surfaces, characteristic of those found on their preferred attachment site on the thorax, whereas *M. nataliae* was more specialized for attachment to hairy surfaces, characteristic of their preferred attachment site on the abdomen. *Poecilochirus carabi* detachment forces (movie S1) were highest on the smooth substrate at all angles, but lowest for the nano/micro-rough surface (smallest asperity size; 0.05 µm) and increasing for larger asperities (0.5 µm) up to the coarse rough surface (16 µm). For the coarse rough (16 µm) and smooth substrates, forces were lower for 90° pull-offs than for 0° and 15° (figure 2*c* and electronic supplementary material, table S5). By contrast, *M. nataliae* detachment forces (electronic supplementary material, movie S2) on artificial surfaces were very low throughout for 90° pull-offs, but for 0° and 15° pulls, forces were highest on the coarse rough (16 µm) substrate, consistent with the use of claws (figure 2*d* and electronic supplementary material, table S5). Compared with *P. carabi*, the pads of *M. nataliae* seemed to be relatively ineffective in gripping smooth substrates.

We measured the force required to detach mites from different sites on the burying beetles. The two mite species differed in their detachment forces on different beetle body regions, but the effect also depended on the pulling angle (GLMM, beetle body part × species × angle: *χ*^2^ = 29.73, d.f. = 3, *p* < 0.001; figure 2*e,f*; electronic supplementary material, tables S6 and S7 for all pairwise *post-hoc* comparisons).

In *P. carabi*, detachment forces for 90° pull-offs were highest on the thorax (figure 2*e*; electronic supplementary material, table S7A; movie S3). However, detachment forces were uniformly high and showed no significant variation between body parts for 15° pulls (figure 2*e*; electronic supplementary material, table S7A). While detachment forces on the beetle's abdomen, elytra and pronotum were significantly lower at 90° than at 15°, this was not the case on the hairy thorax (electronic supplementary material, table S7B). Since *P. carabi* mites were able to deform their arolium around the hairs (see above, electronic supplementary material, movie S4), a 90° pull resulted in the arolia being pulled along the hairs, similar to a low-angle pull on a smooth surface.

For *M. nataliae*, the detachment forces were clearly maximal on the abdomen (and larger than on all the artificial rough substrates) both for the 15° and 90° pulling angles (electronic supplementary material, table S7A). Interestingly, while 90° pull-offs resulted in lower detachment forces on the other body parts, they produced even higher forces on the abdomen (electronic supplementary material, table S7B; movie S5), resulting in extremely high safety factors (5140 ± 2570; mean ± s.d.; *n* = 12).

## 4. Discussion

Our experiments demonstrate that biomechanical adaptations, associated with different attachment sites, can facilitate spatial niche partitioning of two mite species coexisting on the same burying beetle host. Specifically, *P. carabi* and *M. nataliae* preferred the body parts where they were able to attach most strongly, using different attachment mechanisms.

Mites on field-collected beetles showed clear spatial niche partitioning, which was confirmed by experiments on mites released on previously mite-free beetles in the laboratory: *P. carabi* attached mainly to the ventral side of the beetle's thorax, whereas *M. nataliae* attached almost exclusively to the ventral side of the beetle's abdomen. However, the distribution patterns of *P. carabi* differed slightly between field and laboratory in that there were proportionally more *P. carabi* on the thorax of field-collected beetles (figure 3). A possible explanation is that during the laboratory experiment, both the ventral and dorsal sides of the beetle were exposed to light. In addition, mites in the laboratory experiment had less time to associate with the beetles. These factors are not mutually exclusive and both could simultaneously contribute to the variation of *P. carabi* distribution observed between field and laboratory settings. Experiments which varied mite densities of each species independently, showed a marked effect of intraspecific competition at high densities for both *P. carabi* and *M. nataliae*. Furthermore, relatively more *M. nataliae* moved to less preferred body parts at higher densities, suggesting that intraspecific competition affects the spatial distribution more strongly in *M. nataliae* than in *P. carabi* (figure 4). Conversely, interspecific competition affected the distribution of *P. carabi*, notably reducing their distribution on the thorax, which is their preferred body part. However, interspecific competition had a negligible effect on the distribution of *M. nataliae* on their preferred body parts (figure 4).

On field-caught beetles, the density of *P. carabi* was on average an order of magnitude greater than the density of *M. nataliae*, which naturally occurs at relatively low density on burying beetles. The marked difference in density between mite species may be partly attributed to interspecific competition for space on burying beetles. Alternatively, or as well, the different reproductive strategies pursued by each species could also account for this difference. *Poecilochirus carabi* travel as deutonymphs on the beetle, and moult into adult males and females that mate only upon arrival at the carrion to be used for reproduction [28]. By contrast, *M. nataliae* travel as fertilized adult females [29], and so can produce offspring even if transported at very low densities. Furthermore, intraspecific competition also appears to impair reproductive success to a greater extent in *M. nataliae* than in *P. carabi* (electronic supplementary material, figure S3). Since the next generation of mites leaves the carrion breeding site on the adult beetles, this might explain why *P. carabi* is typically an order of magnitude more abundant on field-caught beetles than *M*. *nataliae* (figure 3*a,c*).

The observation that *P. carabi* use their adhesive pads, whereas *M. nataliae* attach by gripping beetle hairs with their chelicerae, suggests that the mites' attachment devices are adapted to specific beetle body regions, which differ substantially in their surface topography (electronic supplementary material, figure S2). Hairs are present on both the thorax and abdomen, but their density is higher on the abdomen. The higher hair density likely makes it more difficult for the mites to reach the beetle's smooth cuticle surface and adhere to it. In fact, dense hairy substrates (e.g. trichome-covered plant leaves) can be challenging for arthropods to navigate and adhere to [30,31] (but see [32–34]), because proper attachment to these surfaces tends to be hampered by the complex topography. However, *P. carabi* seems to cope well with this hairy substrate, despite not having claws that are often involved in strong attachment of other arthropods [35,36]. Nevertheless, *M. nataliae* showed a strong preference for attaching to the abdomen, and specifically targeted the long hairs located on the anterior side of the 1st abdominal sternite. Thus, the differences in the attachment devices of *P. carabi* and *M. nataliae*, and the heterogeneity of the beetle's body surface may explain how the two mite species partition the space on their host.

Phoretic mites possess diverse structures for attaching to their hosts including claws, adhesive pads, anal pedicels and chelicerae [37,38], but the substrate-specific performance and ecological significance of these mechanisms was previously unknown. Our pull-off force measurements on different substrates and beetle body parts confirm that the mites are biomechanically adapted to different body parts of their host. In *P. carabi*, the high shear forces (angles 0° and 15°) on the coarse rough (16 µm) substrate, despite their lack of claws, are notable because previous studies of insect adhesive pads showed no or only little increase of attachment force for larger asperities [39,40]. The high forces required to shear *P. carabi* may be based on the small size of their pads, allowing them to find relatively flat contact regions on a coarse rough substrate [40,41]. The relatively high attachment forces of *P. carabi* at pull-off angles 15° and 90° on the hairy thorax further suggest that *P. carabi* also copes well with hairy substrates, despite their lack of claws, which are often involved in strong attachment in other arthropods [32,33]. The attachment of *P. carabi* was less direction dependent on the thorax than on the other body parts, and also less direction dependent than typical attachment systems of climbing arthropods [32,33]. This unusual performance can be explained by the fact that the arolium of *P. carabi* mites can adhere by wrapping itself around a single beetle hair (electronic supplementary material, movie S4).

*Macrocheles nataliae* achieved even higher detachment forces on the hairy beetle substrates for oblique (15°) and especially perpendicular (90°) pull-offs. However, they produced these very high forces only on the beetle's abdomen but not on the other body parts. This indicates that *M. nataliae* mites cling to the abdomen with a different attachment mechanism, consistent with our video observations that they use their chelicerae to grip the abdominal hairs. The attachment forces of *M. nataliae* on the beetle abdomen exceed even the extreme values reported for mites by Heethoff & Koerner [35], which illustrates the effectiveness of the pincer grip on the hairs. It is remarkable that, although hairs are also present on the beetle thorax, *M. nataliae* rarely, if ever, managed to grip them with their chelicerae. This is likely because the hairs on the thorax are too thin to allow the chelicerae to grip them. In particular, the two gaps on both sides of the large tooth on the movable chelicera segment in *M. nataliae* (figure 5*d*) are hemicircles of *ca* 8–10 µm radius. Thus, it is likely that thinner hairs will slide through the gaps even at full closure. Whether *M. nataliae* chelicerae are indeed specialized to grip hairs of a specific diameter remains to be tested experimentally. It is also noteworthy that the preferred location of *M. nataliae* on the abdomen may be more shielded from mechanical disturbance and drag forces (figure 1*a,c*) when the host beetle moves forward through the substrate, grooms its body surface or flies in search of a new carcass [42].

The two mites’ adaptations for clinging to their host burying beetles can be compared with adaptations of ectoparasite insects that attach to body hairs or feathers of mammals, birds or other arthropods. The chelicera-based attachment mechanism of *M. nataliae* is similar to that of some ectoparasite insects, which use dentate or comb-shaped claws to interlock with host hairs [32,33]. A key difference is that the ectoparasites' attachment is passive and likely more energy efficient than the active grip of *M. nataliae.* While such passive interlocking makes the detachment from the hair more difficult or slower [33], the active grip of *M. nataliae* and the adhesion-based strategy used by *P. carabi* probably facilitate locomotion on the host, or detachment from it.

In conclusion, our findings show that *P. carabi* and *M. nataliae* mites differ in their reproductive output, density and preferred site of attachment while travelling on their burying beetle host. Their spatial separation on the beetle host can be explained by biomechanical adaptations that enable *P. carabi* and *M. nataliae* to attach safely on their preferred sites. It is possible that interspecific competition originally drove this trait divergence, but we found little evidence to suggest that competition currently exists between the two mite species. Therefore, it is just as possible that trait divergence between the mite species preceded their joint association with the burying beetle and is not due to competition. Comparative analyses involving many more mite species are needed to distinguish between these alternative scenarios.

## Data Availability

The data and code have been deposited in the Dryad Digital Repository (https://doi.org/10.5061/dryad.41ns1rnnc) [43]. Supplementary material is available online [44].
